# A Text-Book of Pharmacology

**Published:** 1902-05

**Authors:** 


A TEXT-BOOK OF PHARMACOLOGY. Including Therapeutics, Materia Medica, Pharmacy, Prescription-Writing, Toxicology, etc. By TORALD SOLLMANN, M. D., Assistant Professor of Pharmacology and Materia Medica, Western Reserve University, Cleveland, Ohio. Royal 8vo volume of 880 pages, fully illustrated. Philadelphia and London: W. B. Saunders & Co. 1901. [Price, cloth, $3.75 net.]

This work aims to furnish, in a manner suited for reference and study, a scientific discussion and definite conception of the action of drugs, as well as their derivation, composition, strength, and dose. The author bases the study of therapeutics on a systematic knowledge of the nature and properties of drugs, and thus brings out forcibly the intimate relation between pharmacology and practical medicine.

The work is divided into four parts: Part I., dealing with the preparation and prescribing of medicines, and toxicologic analysis; Part II., with pharmacology, therapeutics and materia medica; Part III., with practical exercises and Part IV., with an appendix. In Part I. the author goes over the ground which beginners in pharmacy and dispensing physicians must follow, describing the gross anatomy and chemistry of plants, pharmaceutical methods, special pharmaceutical preparations and gives a very important and interesting chapter on prescription writing. The different flavoring methods are thoroughly described, as are the combinations which appeal to the taste as well as to the eye.

Under Part II., the author takes up those drugs whose main action occurs after absorption, and also those drugs whose action is a local one. These chapters comprise the bulk of the work and each drug is thoroughly dealt with in its different medical phases. Part III., is a laboratory guide and deals with the methods in vogue, effects of drugs and animal experiments. Part IV.,, the appendix, takes up the methods of analysing the causes of pharmacologic action, dose table and drug index.

Practitioners and students will find the work an admirable guide in that most important part of their equipment—namely, how to use drugs accurately and efficaciously. The book will also be of the utmost service, not alone to students and practitioners, but also to druggists and everyone interested in the use of medicines. W. C. K.

